# Active power control strategy for wind farms based on power prediction errors distribution considering regional data

**DOI:** 10.1371/journal.pone.0273257

**Published:** 2022-08-24

**Authors:** Mst Sharmin Kader, Riyadzh Mahmudh, Han Xiaoqing, Ashfaq Niaz, Muhammad Usman Shoukat

**Affiliations:** 1 College of Electrical and Power Engineering, Taiyuan University of Technology, Taiyuan, China; 2 Shanxi Key Laboratory of Power System Operation and Control, Taiyuan University of Technology, Taiyuan, China; 3 Hubei Key Laboratory of Advanced Technology for Automotive Components, School of Automotive Engineering, Wuhan University of Technology, Wuhan, China; University of Wolverhampton, UNITED KINGDOM

## Abstract

One of the renewable energy resources, wind energy is widely used due to its wide distribution, large reserves, green and clean energy, and it is also an important part of large-scale grid integration. However, wind power has strong randomness, volatility, anti-peaking characteristics, and the problem of low wind power prediction accuracy, which brings serious challenges to the power system. Based on the difference of power prediction error and confidence interval between different new energy power stations, an optimal control strategy for active power of wind farms was proposed. Therefore, we focus on solving the problem of wind power forecasting and improving the accuracy of wind power prediction. Due to the prediction error of wind power generation, the power control cannot meet the control target. An optimal control strategy for active power of wind farms is proposed based on the difference in power prediction error and confidence interval between different new energy power stations. The strategy used historical data to evaluate the prediction error distribution and confidence interval of wind power. We use confidence interval constraints to create a wind power active optimization model that realize active power distribution and complementary prediction errors among wind farms with asymmetric error distribution. Combined with the actual data of a domestic (Cox’s Bazar, Bangladesh) wind power base, a simulation example is designed to verify the rationality and effectiveness of the proposed strategy.

## 1 Introduction

After years of rapid development in Bangladesh, wind power has entered the platform period, and the problem of wind power adaptability in the power grid is becoming more and more prominent. Large scale wind power grid connection increases the pressure on the secure and steady operation of the power system. Therefore, improving the active support performance of wind power generation to the power grid and reducing the impact of its forecast deviation on the active power balance control has become the core problem of wind power generation system [1].

In order to realize the active support of wind power generation system to the power grid, wind power station needs to have good measurement accuracy, control performance and regulation ability like traditional power supply. Firstly, the power forecast level of wind power station needs to be greatly improved to meet the accuracy requirements of power grid dispatching operation; secondly, under the premise of steady operation of the power grid, it can automatically adjust the power output of the wind power station to meet the demand of the power grid; finally, it is necessary to make rapid adjustments in response to the changes in the operational state of the power system [2, 3].

Wind farm active power control has a connecting role in the active power dispatching system including large-scale wind power farms [4]: it not only tracks the output plan of wind farms, but also distributes the cluster dispatching instructions to the units in the wind farm to coordinate and control the active power output of each unit in the field. Most of the research on the active power control strategy of the existing wind farm takes the minimum sum of the internal power loss of the wind farm as the optimization objective [5], and also considers the active power control of wind power prediction technology. The control objective is mainly to reduce the deviation of active power distribution and realize the stable output of wind farm power [6]. Therefore, the active power control of wind farm should not only take the power loss as the optimization object, but also fully tap the active power regulation ability of wind farm. Wind speed and wind power forecasting’s main purpose is to provide information on predicted wind speed and power over the next several minutes, hours, or days. The prediction can be separated into four timeframes based on power system operation needs [7]: ultra-short-term (a few seconds to 4 h), short-term (4 h to 24 h), medium-term (1 to 7 days), and long-term (more than 7 days). Turbine control and load tracking are done with ultra-short-term predictions, whereas power system management and energy trading are done with short-term forecasts, and wind turbine maintenance is done with medium-term and long-term forecasts [8].

At present, there have been many research results in the power control of wind power plants, which can be unevenly divided into two classes: proportional distribution method and optimal distribution method [9, 10]. In order to improve overall performance, a feature extraction-based data pre-process strategy is proposed in [11] to reduce wind power generation fluctuations and select suitable input forms of wind speed datasets, as well as an uncertain set model selection procedure to fix the best cooperation solutions from the Pareto front set derived from the optimization stage. Based on the operating characteristics of doubly-fed wind turbines, literature [12] proposed a wind farm power distribution method according to the maximum available power of the generating units. Literature [13] approved the optimal allocation method, sets different optimization objectives, constructs the objective function, and realized the decomposition of power control objectives. A unit commitment optimization model for wind farms, and the power allocation optimization model of wind turbine in wind farm proposed in [14] to introduce the model predictive control and frequency constraints to improve the optimization model. Author in [15] proposes a state classification model based on wind turbine based on the changes of wind turbine operation state in adjacent regulation periods to realize the smooth output of wind farm power. Authors in [16] introduce the research and development of wind power control system and its application in practical engineering. Author in [17] developed a new energy power control system suitable for large-scale wind power generation base, which decomposes the power generated by the wind farm in the base with section and line stability constraints according to the wind power forecast and power generation instructions issued by the power grid. However, the proposed model’s prediction accuracy depends on regression ability and decrease with large sample. By combining several optimization methods, the boosting method can improve the fundamental model’s ability.

Author in [18] implements a novel hybrid deep learning-based evolutionary technique to improve wind speed forecast accuracy. Literature [19] used a re-analysis of the Modern-Era Retrospective Analysis for Research and Applications version 2 (MERRA-2) to recognize long-term Mediterranean Sea Offshore Wind (OW) arrangement probable settings, as well as machine learning tactics based novel combined intelligent to forecast OW speed. To decompose signals and pre-processing data, evaluate the upcoming total of wind turbines energy production, and optimize the fuzzy GMDH neural network parameters, literature [20] proposed a mutual prediction model based on empirical mode decomposition, fuzzy group method of data handling neural network, and grey wolf optimization algorithm. Author in [21] provides an innovative adaptive neuro-fuzzy inference method to estimate the yield power of a wind turbine based on wind power inputs such as wind speed, turbine rotational swiftness, and mechanical-to-electrical power converter temperature.

The interval prediction is calculated using the point prediction result and the confidence interval for the mistakes. The upper and lower bounds of the errors’ confidence interval are added to the point prediction results, and the interval estimate result is derived at a specific confidence level. Interval predicting, as opposed to point prediction, can provide more quantitative data about the wind power generation uncertainty [22, 23]. As determent by the error among the point prediction result and the actual wind power, a confidence interval optimization method-based wind power interval forecasting is used in [24] to analyze the lowest confidence interval length of the random shape distribution. Moreover, the current confidence interval calculation method is only valid for a given distribution.

**Fig 1:**
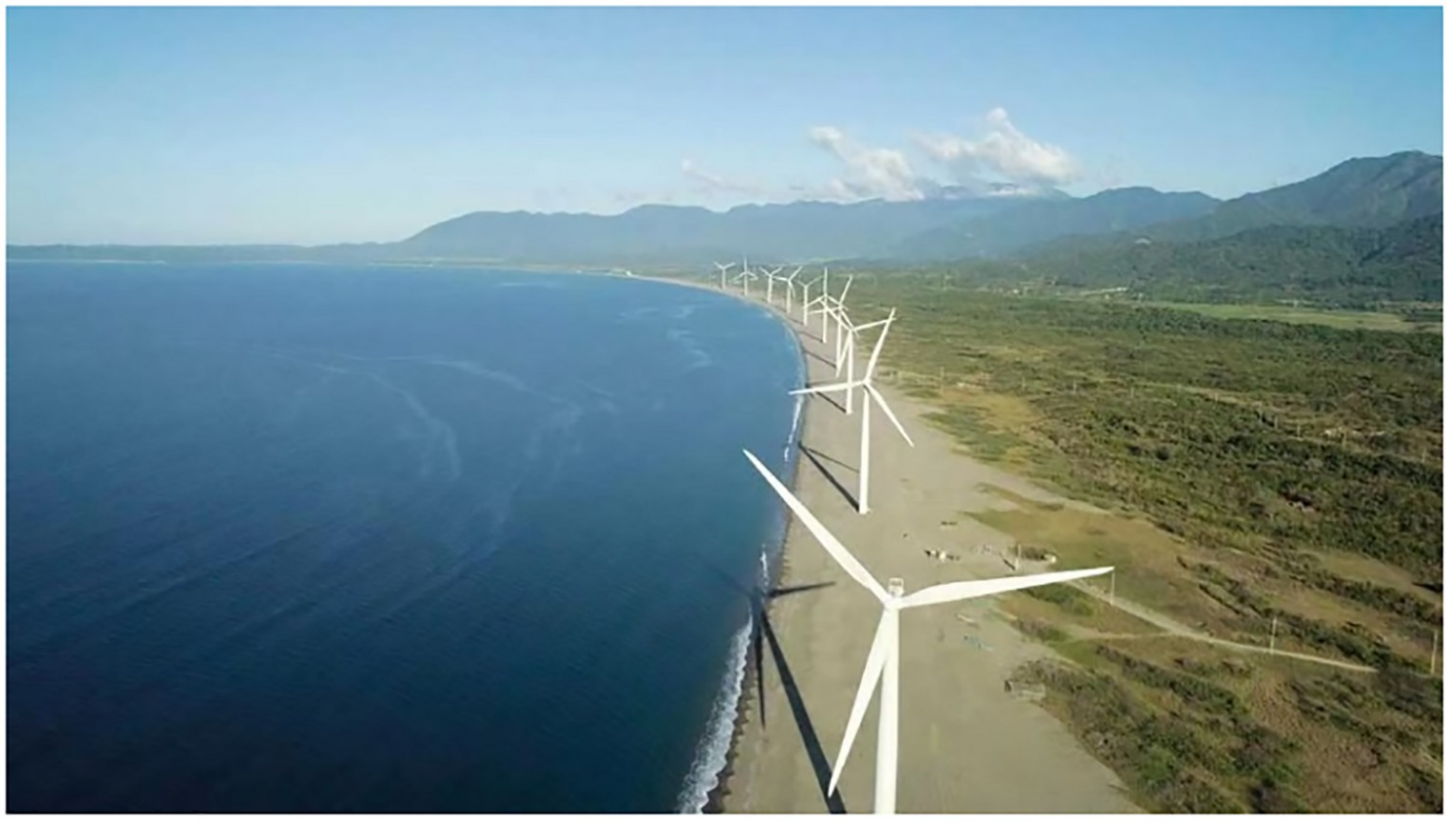
Wind power farm of Cox’s Bazar Bangladesh.

The above research considers the forecast power of wind power as relatively certain. However, the wind resource itself has random fluctuation, and there are inherent errors in the forecast power, which poses a challenge to the accurate control of the active power of the wind power station. Therefore, when formulating the optimal control of wind power generation, it is compulsory to practically consider the forecast error [25]. In the actual operation process, due to the relative 3D dispersion and different technical differences of wind power plants, the operating characteristics of wind power stations, and the error distribution characteristics are significantly different [26]. Accurately grasping the operation characteristics and power forecast error distribution characteristics of new energy is of great significance for improve the power control level of new energy sources. Wind power forecast methods can be sorted into four basic methods: time series model, machine learning model, deep learning, and combined forecast model. Table 1 summarizes the advantages, disadvantages and applicability of four categories and thirteen common wind power forecast methods, as well as Fig 1 shows the wind power farms of Cox’s Bazar under Bangladesh power development board [27]. The gaps are as follows to highlight the main issues in developing active power control forecasting models:

- The random parameter tuning optimization method has an important influence on the performance of the active power control model. In general, the metaheuristics used to change the random parameters are inefficient, while initializing the control limits is complicated and time-consuming. Also, the majority of them were constructed using numerical benchmarks rather than random constraint tuning on the target problem.
- In order to minimize the economic loss caused by the control error through the wind power forecast error, the confidence interval as an optimization model plays a significant role. This collection procedure might be difficult since improper setting selections can have a negative impact on the forecasting models’ performance.

**Table 1:** Forecasting methods and characteristics of wind power.

Category	Method	Advantages, disadvantages, and applicability	Literature
Time series	Continuous method	When the calculation is simple, it is only suitable for ultra-short-term prediction, and the fluctuation of wind power is not drastic, the error is the smallest.	[28–30]
	ARMA	The calculation is relatively simple, suitable for stationary time series.	[31]
	ARMIA	When smoothing nonlinear data, it is hard to determine the optimal structural constraints for strong nonlinear data.	[32]
Machine learning	SVM	Higher order (1–11 orders) can improve the forecast effect, but the kernel parameters and penalty factors of SVM are difficult to choose, so optimization algorithm is generally used to determine them.	[17, 33, 34]
	RF	It has strong robustness to non-important influencing factors and noise data, and has satisfactory results without optimizing structural parameters.	[35, 36]
	GP	It has strong generalization ability for nonlinear and small sample data.	[37]
Deep learning	BP	The neural network based on error back propagation is generally used as the benchmark model.	[38, 39]
	ELM	The number of hidden units only needs to be set faster.	[40]
	CNN	It has a strong capability to extract the implicit connection features of the data, and adopts the weight parameter sharing technology to reduce the difficulty of model training.	[41]
	RNN	It can process complex time series and can mine the feature relationship of data in the time dimension well, but RNN is easy to train in the model	[42, 43]
	LSTM, GRU	LSTM and GRU solve the phenomenon of long-term dependence to a certain extent.	[44]
Combined forecast	Data decomposition	The nonlinear and non-stationary wind speed or wind power data is processed to reduce the difficulty of training; it has strong generalization ability and forecasting accuracy.	[45]
	Weight coefficients	According to the characteristics of different algorithms, it recovers the robustness of the prediction model to a certain range; The combination model based on variable weight coefficient has stronger adaptability than that based on fixed weight coefficient.	[46, 47]

Based on these gaps, we focus on solving the problem of wind power forecasting and improving the accuracy of wind power prediction and will strive to achieve the following innovative objectives:

- In this paper we consider the difference in power forecast error of different new energy power stations, and optimize the active power control of wind power.
- Firstly, the wind power prediction data and actual operation data are collected.
- On this basis, the distribution features of wind farm power prediction errors in different locations and their influence on active power control are analyzed.
- The active power control of wind farms is integrated and optimized based on different error distribution characteristics.
- At the same time, combined with the actual scenarios, we establish a case for method verification in order to prove the method’s effectiveness.

## 2 Analysis of wind power prediction error

### 2.1 Power prediction error distribution

According to the predicted power *P*_*p*_ and the actual power *P*_*a*_ of the wind power station, the absolute error of the predicted power can be calculated. Considering that when the output of the wind farm is small, the small absolute error may cause a large relative error, which is inconvenient for statistical analysis. Therefore, the rated power *P*_*wp*_ of the wind farm is taken as the reference value to calculate the relative error *e* of the wind farm, that is,

$$e=\frac{P_{p}-P_{a}}{P_{wp}}$$ (1)

Two types of hypothesis test methods such as null and alternative hypothesis are conducted in [48] to distinguish the errors for the proposed sachem and the compared model, where the null theory means that there is no change among the prediction errors of compared models and the alternative hypothesis means that the prediction error of the proposed model is lower than compared one. After verification by the hypothesis test method and *χ*^2^ test method in mathematical statistics (more precisely, mean and quantiles), it is found that the test value of the statistic falls in the receptive domain, so the predicted power error of the wind farm obeys the normal distribution.

### 2.2 Power prediction error confidence evaluation model

The evaluation index of power forecast model can quantify the error characteristics of prediction model. The mathematical expressions and application scope of forecast and evaluation indexes are shown in Table 2. In recent years, more scholars and experts have applied the combination of quantile regression and risk assessment to the field of wind power forecasting [49, 50]. The distribution function *F*(*y*) = *P*(*Y* ≤ *y*) can be used to describe the properties of the random variable *Y*, and the *τ* quantile function of *F*(*y*) is defined as:

$$Q_{\left(\tau \right)}=\text{inf}\;\left\{y:F\left(y\right)\geq \tau \right\},\;0<\tau <1$$ (2)

**Table 2:** Power forecast evaluation indicators and their features.

Category	Evaluation models	Mathematical expression	Features
Basic indicators	Absolute Error	$\left|y_{a}^{i}-y_{p}^{i}\right|$	Describes the difference between a single estimate and the actual value [51].
	Relative Error (RE)	$\left|(y_{a}^{i}-y_{p}^{i})/y_{a}^{i}\right|$	Describes the reliability of a single forecast [52].
	Bias	$\frac{1}{N}\sum_{i=1}^{N}\left(\frac{y_{a}^{i}-y_{p}^{i}}{y_{nom}}\right)$	An infinite description of bias, suitable for evaluation between different forecast models [53].
Mean-Based Evaluation	Mean Absolute Error (MAE)	$\frac{1}{N}\sum_{i=1}^{N}\left({|y}_{a}^{i}-y_{p}^{i}|\right)$	Describes the deviation of forecast and actual values, reflecting the overall level of error, suitable for large-scale data evaluation [54, 55].
	Mean Absolute Percentage Error (MAPE)	$\frac{1}{N}\sum_{i=1}^{N}\left(\frac{y_{a}^{i}-y_{p}^{i}}{y_{a}^{i}}\right)\times 100\%$	
	Normalized Mean Absolute Error (NMAE)	$\frac{1}{N}\sum_{i=1}^{N}\left(\frac{y_{a}^{i}-y_{p}^{i}}{y_{nom}}\right)\times 100\%$	
Mean square evaluation index	Root Mean Square Error (RMSE)	$\frac{1}{N}\sqrt{\sum_{i=1}^{N}{\left(y_{a}^{i}-y_{p}^{i}\right)}^{2}}$	Suitable for multi-objective evaluation with less variance by evaluating forecast bias [56].
	Normalized Root Mean Square Error (NRMSE)	$\frac{1}{N}\sqrt{\sum_{i=1}^{N}{\left[(y_{a}^{i}-y_{p}^{i})/y_{nom}\right]}^{2}}$	Suitable for multi-objective evaluation with small variance [57].
	Root Mean Square Relative Error (RMSRE)	$\frac{1}{N}\sqrt{\sum_{i=1}^{N}{\left[(y_{a}^{i}-y_{p}^{i})/y_{a}^{i}\right]}^{2}}$	Evaluate the deviation of forecast and actual values [58].
	Root Mean Squared Logarithmic Error (RMSLE)	$\frac{1}{N}\sqrt{\sum_{i=1}^{N}{\left(log\left(y_{a}^{i}+1\right)-\text{log}\;\left(y_{p}^{i}+1\right)\right)}^{2}}$	It is suitable for the situation where the forecast value and the actual value are too different at a certain moment [59].
Other evaluation indicators	Improve Mean Absolute Error (IMAE)	|(*E*_*MAE*1_ − *E*_*MAE*2_)/*E*_*MAE*1_|	It is suitable for evaluating the forecast effect between different models [60].
	Improve Root Mean Square Error (IRMSE)	|(*E*_*RMSE*1_ − *E*_*RMSE*2_)/*E*_*RMSE*1_|	
	Mean Trend Deviation (MTD)	$\frac{1}{y_{nom}(N-1)}\sum_{i=2}^{N}\left(\Delta y_{a}^{i}-\Delta y_{p}^{i}\right)$	It is suitable for power forecast to evaluate the stability of power grid [61, 62].
	Friendship (F)	$\sum_{i=1}^{N}\left|\left(e_{a}^{i}-e_{p}^{i}\right)-k\left(y_{a}^{i}-y_{p}^{i}\right)\right|/N$	
Uncertain forecast	Average Coverage Error (ACE)	*PICP* − *PINC*	Describes the reliability of prediction intervals, suitable for small-scale data [63].
	Prediction Interval Reliability	$\sum_{i=1}^{N}C_{i}/N$	Reflecting the reliability and quality level of the predictive model is a necessary condition for the uncertain forecasting of wind power [64].
	Prediction Interval Average Width	$\sum_{i=1}^{N}(U_{i}-L_{i})/N$	
	Normalized Prediction Interval Average Width	$\sum_{i=1}^{N}\left(U_{i}-L_{i})/(N\times y_{nom}\right)$	Reflects the overall width of the forecast interval, suitable for large-scale data [65].

Note: *y*_*a*_, *y*_*p*_, and *y*_*nom*_ are divided into actual power, forecast power, and rated power value of wind power, respectively. *y*_*a*_ is the actual average power of the test data set; *N* is the length of the test data set, *e*_*a*_ and *e*_*p*_ are divided into the actual load value and the forecast load value of wind power, if *y*_*a*_ ∈ [*L*_*i*_, *U*_*i*_] then *C*_*i*_ = 1, otherwise *C*_*i*_ = 0, *L*_*i*_ and *U*_*i*_ are the upper and lower boundaries of the forecast power interval, *I*_*i*_ = [*L*_*i*_, *U*_*i*_]. *CDF*_*i*_ is the given cumulative distribution function; if *y* < *y*_*i*_, then *H*(*y* − *y*_*i*_) = 0; otherwise *H*(*y* − *y*_*i*_) = 1.

It can be known from Eq (2) that the proportion of variables smaller than the quantile function *Q*_(*τ*)_ is *τ*, and the proportion of variables larger than the quantile function *Q*_(*τ*)_ is (1 − *τ*). Define the inspection function as:

$$\rho \left(u\right)=\tau uf\left(u\right)+(\tau -1)uf(u)$$ (3)

where, *f*(*u*) = 0 when *u* ≥ 0, and *f*(*u*) = 1 when *u* < 0. Let *u* = *y* − *δ*, take the expectation on the both sides of the above equation, and then take the derivative of *δ*:

$$F\left(\delta \right)=\left(1-\tau \right)\int_{-\infty }^{\delta }dF\left(x\right)-\tau \int_{\delta }^{+\infty }dF\left(x\right)=0$$ (4)

Since *F* is monotonically increasing, an element in the set {*y*: *F*(*δ*) = *π*} can be found to minimize *E*(*ρ*(*y* − *δ*)) in any interval. Defined by the quantile *Q*(*τ*│*x*) = *x*’ *β*(*τ*), when any random parameter satisfies min_*β*∈*R*_ ∑_*i*_ *ρτ*(*y*_*i*_ − *x*’*β*(*δ*)), solve arg min_*β*∈*R*_ ∑_*i*_ *ρτ*(*y*_*i*_ − *x*’*β*(*δ*)), $\hat{\beta }(\delta )$ can be obtained.

### 2.3 Confidence assessment of power forecast

Given quantile (*τ*_1_, *τ*_2_, ⋯, *τ*_*n*_), a quantile regression module of wind power forecast is established, and then combined with the likelihood distribution and confidence level of wind power error, the confidence interval of wind power forecast error can be found [66], and then the confidence interval of wind power forecast can be gained. Due to the dissimilar power forecast models and linked manipulating features, the forecast error distribution of different wind farms shows obvious asymmetry over a period of time [67, 68]. Among them, some wind farms’ forecast power is very close to the lower boundary of the confidence interval, indicating that the possibility that the wind farm’s actual available power is larger than the predicted power is high, and wind farms with similar confidence intervals are noted as Ω_+_. Some wind farms’ predicted power is very close to the upper boundary of the confidence interval, indicating a high possibility that the wind farm’s actual available power is less than the predicted power, and wind farms with similar confidence intervals are reported as Ω_−_.

## 3 Framework

Usually, the active power of the wind farm is controlled only when the forecast power of the wind farm is greater than the power generation plan. The active power control demand Δ*P*_*i*_ of wind farm *i* is determined by the forecast power and generation plan:

$$\Delta P_{i}=P_{d,i}-P_{p,i}$$ (5)

where, *P*_*d*,*i*_ and *P*_*p*,*i*_ are the power generation plan and predicted power of wind farm *i*, respectively.

When implementing the active power control of the wind farm, the active power adjustment amount undertaken by the Ω_+_ type wind farm is Δ*P*_+_, and the active power adjustment amount undertaken by the Ω_−_ type wind farm is Δ*P*_−_.

The output power Δ*P*_+_ of the Ω_+_ type wind farm *i* should be;

$$P_{i+}=P_{p,i+}+\frac{P_{N,i+}}{\sum_{i\in \Omega_{+}}P_{N,i+}}\Delta P_{+}$$ (6)

where, *P*_*p*,*i*+_ and *P*_*N*,*i*+_ are the predicted power and installed capacity of the Ω_+_ type wind farm *i*, respectively.

The output power Δ*P*_−_ of the Ω_−_ type wind farm *i* should be;

$$P_{i-}=P_{p,i-}+\frac{P_{N,i-}}{\sum_{i\in \Omega_{-}}P_{N,i-}}\Delta P_{-}$$ (7)

where, *P*_*f*,*i*−_ and *P*_*N*,*i*−_ are the predicted power and installed capacity of the Ω_−_ type wind farm *i*, respectively.

In actual operation, the high probability of output power of the Ω_+_ type wind farms are likely to meet the control target, while the high probability of output power of the Ω_−_ type wind farms is likely to be lower than the control target, which will source the active power control of wind farm set to fail to meet the dispatching instruction.

## 4 Methodology

### 4.1 Optimization algorithm

According to the principle, optimization algorithms can be divided into four categories, such as optimization algorithms based on evolutionary assumed, group based social intelligence, physical supposed and geographical assumed [69], and their typical algorithms and characteristics are shown in Table 3.

**Table 3:** Types and characteristics of optimization algorithms.

Classification	Algorithm	Features
Evolutionary assumed	Genetic Algorithms [53], and Differential Evolution Algorithms [70],	By simulating the principle of biological evolution, individuals not only have a strong ability to meet the environment, but also pass this ability to offspring, but sometimes it is easy to fall into a local optimal solution.
Social intelligence	Particle Swarm Algorithm [71], and Fruit Fly Optimization Algorithm [72]	Although the individuals in the group are relatively simple, they can provide concise, fast and effective solutions to complex problems through cooperative collective behavior.
Physical supposed	Gravity Search Algorithm [73], Water Cycle Algorithm [74], and Atomic Search Algorithm [75]	It follows the laws of physics in the physical world, and its ideas are concise and easy to understand. It is generally used in combination with other algorithms to achieve global optimization.
Geographical assumed	Avoidance Search Algorithm [76], Imperialist Competition Algorithm [77], and Biogeographic Optimization Algorithm [78]	It is simple and easy to implement, but it is informal to fall into an extreme point, and global optimization cannot be guaranteed.

**Fig 2:**
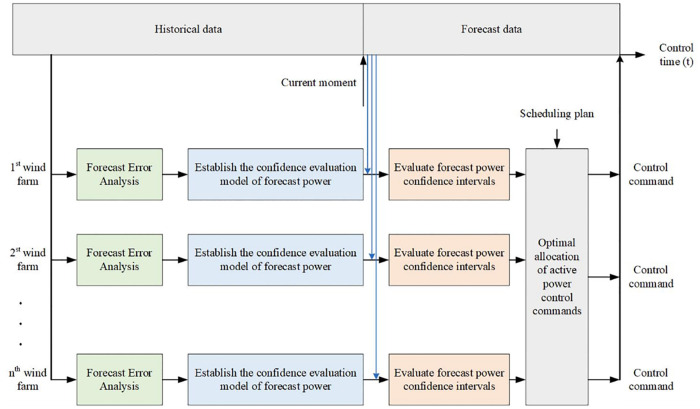
Wind power active power control process.

In this paper, according to the historical power forecast error data, we are statistically analyzed the error distribution characteristics, and the method of section 2.2 is used to establish the confidence evaluation model of the forecast power. On this basis, combined with the ultra-short-term power forecast data, if the forecast power curve is corrected to the predicted power band, then the output of the wind farm should be in the predicted power band. In order to minimize the economic loss caused by the control error through the wind power forecast error, the confidence interval as an optimization model is used as a constraint to give the active power control model. It takes the ultra-short-term power forecast data and dispatching directions as input and optimizes the active power control commands of each wind farm. The specific process is shown in Fig 2.

### 4.2 Active power control model

Gu et al., [79] according to the distribution features of wind power forecast error, the expectation of different wind farms can be analyzed and calculated, and then the power forecast expectation *P*_*E*,*i*_ is;

$$P_{E,i}=P_{p,i}+\int_{0}^{e_{\tau }}e∙F_{i}(e)de$$ (8)

where, *P*_*p*,*i*_ is the predicted power of wind farm *i*; *F*_*i*_(*e*) is the power forecast error probability distribution function of wind farm *i*.

The objective function is to minimize the change among the active power and the predicted power of the wind farm as [4];

$$minf=|\sum \left(P_{i}-P_{E,i}\right)|$$ (9)

Constraints are;

∑Pi=PdPmin≤Pi≤Pmax (10)

where, *P*_*i*_ is the output power command of wind farm *i*.

The first constraint specifies that all wind farms’ active power must be consistent with the power generation plan and the aggregate of all wind farm power outputs must fulfill the scheduling directions. The second is the wind farm operation constraint, that is the limit constraint of wind farm *i*. The confidence interval lower limit of the power calculation and the higher value of the minimum operating power of the wind farm must be the minimum power yield of the wind farm. The wind farm’s maximum power output should be equal to the power prediction’s upper confidence interval and the lower value of the wind farm’s minimum operating power.

## 5 Overview results analysis

### 5.1 Example overview

**Fig 3:**
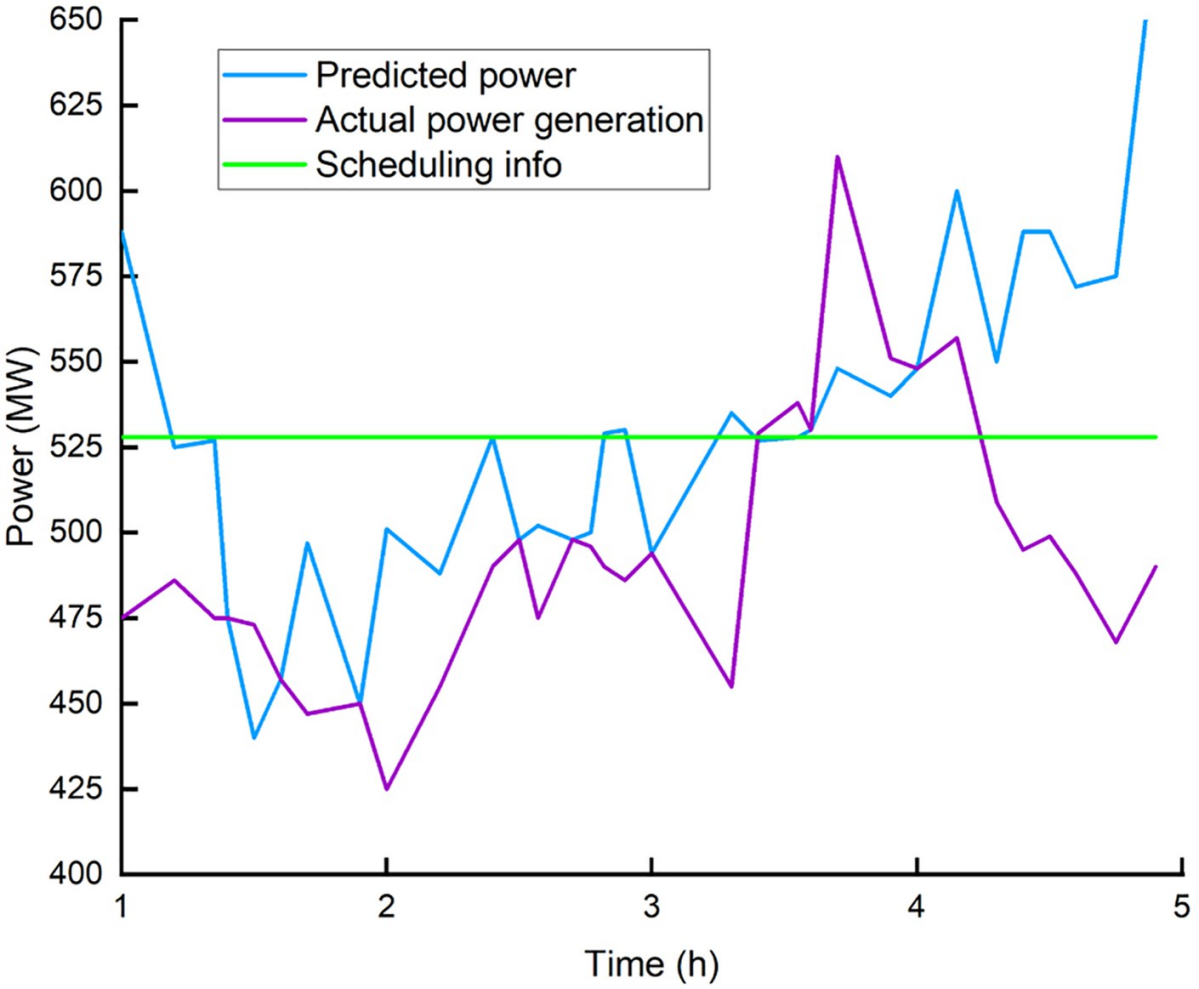
Data of wind power and generating schedule. (a) 1^st^ wind farm, (b) 2^nd^ wind farm, (c) 4^th^ wind farm.

Based on the predicted power of four wind farms (with a total installed capacity of 903MW) in a domestic (Cox’s Bazar, Bangladesh) wind power base from January to July 2021 and measured the regional power data after theoretical reduction, in this paper, we compare and verify the control effect of this method. Part of the data is used to examine the wind farm’s predicted power distribution characteristics, while the rest is utilized to compare and validate the proposed strategy’s effect. Fig 3 shows the forecast power, measured power and power generation commands of the wind farm.

**Fig 4:**
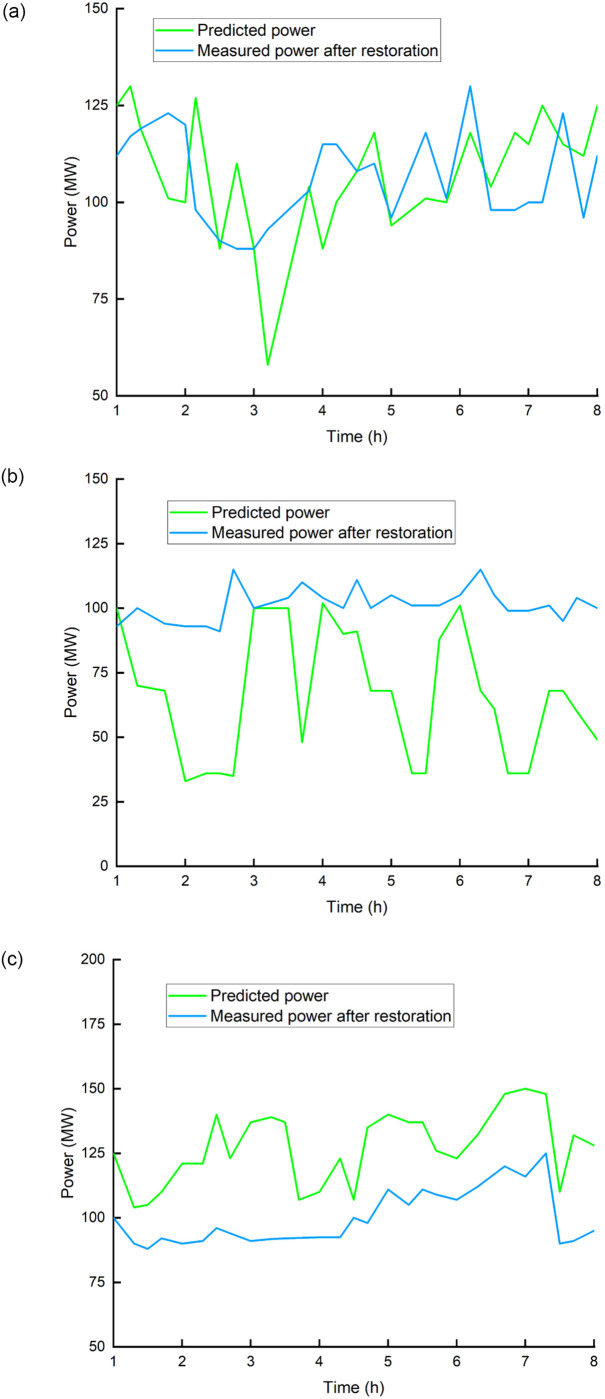
Farecasting and measured power output of wind farms. (a) Actual control effect, (b) Control deviation comparison.

The installed capacity of the four wind farms is as: the 1^st^ wind farm 201MW; the 2^nd^ wind farm 201MW; the 3^rd^ wind farm 300MW; and the 4^th^ wind farm 201MW. Fig 4 shows the predicted power of certain wind farms such as 1^st^, 2^nd^, and 4^th^ in the corresponding period of the day, as well as the measured power after theoretical reduction.

It can be seen from Fig 4, the1^st^ wind farm’s measured power is near to the forecast power, the 2^nd^ wind farm’s actual power generation capacity is better than the forecast power, and 4^th^ wind farm’s actual power generation capacity is lower than the forecast power. Set the confidence level to 0.95. Calculate the confidence interval for each wind farm’s predicted power, That is evidently irregular. It can be realized that the positive and negative error intervals of the 1^st^ wind farm are essentially symmetrical, the 2^nd^ wind farm has positive error characteristics, and the 4^th^ wind farm has negative error characteristics. This example compares the suggested optimization algorithm to the capacity proportional allocation method to examine its control effect within this border.

### 5.2 Results analysis

**Fig 5:**
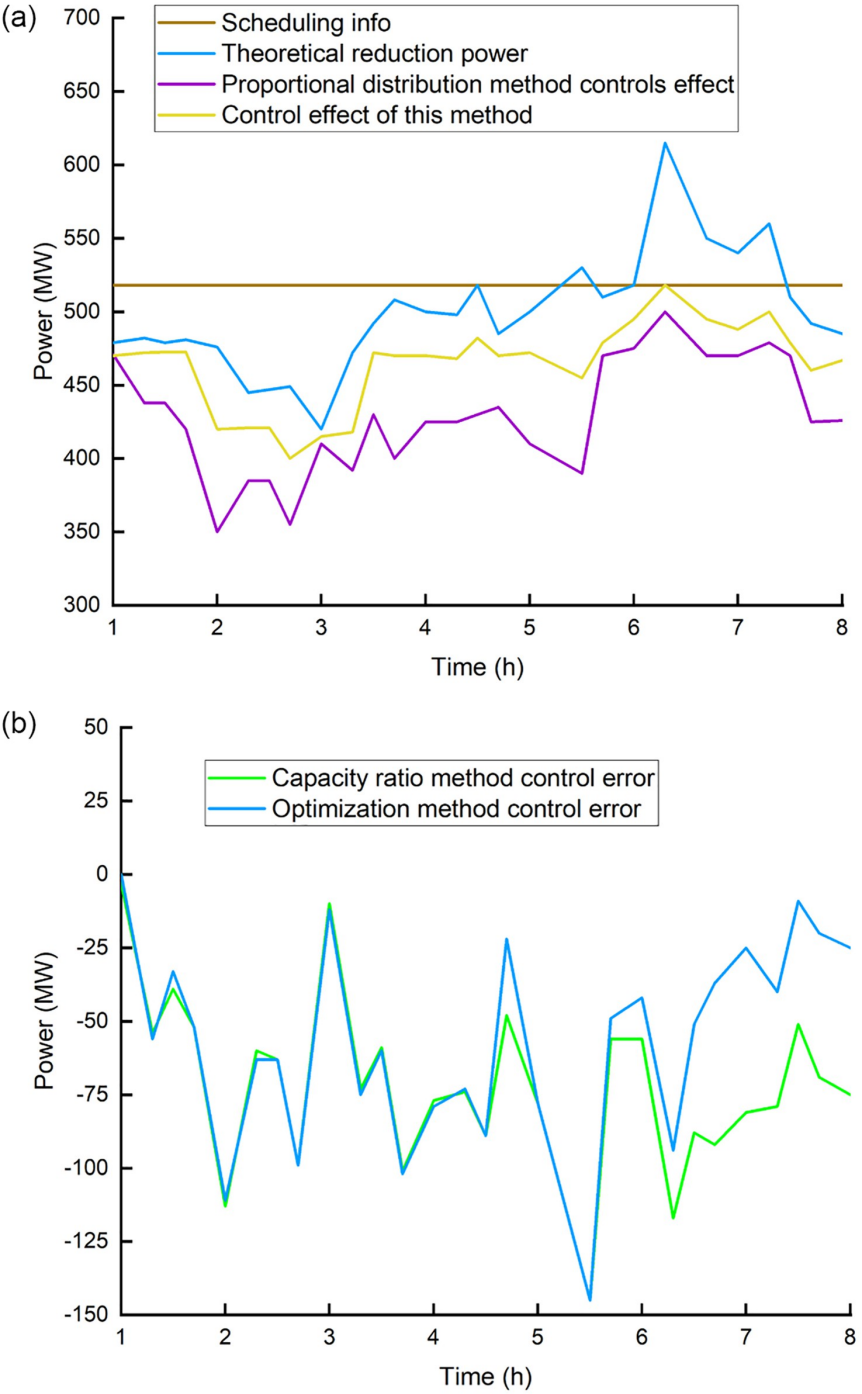
Comparison of the control effects of the two methods. (a) 1^st^ wind farm, (b) 2^nd^ wind farm, (c) 4^th^ wind farm.

For the wind power prediction error distribution, Fig 5 shows a comparison of the proposed approach’s control effect with that of the traditional capacity proportional distribution method.

As can be seen from Fig 5 that the control objective obtained by the method in this paper is more accurate, and the overall control deviation is significantly better than the traditional capacity proportional distribution method. Table 4 shows the probability of the power prediction errors distribution for the three wind farms with normal distribution type.

**Table 4:** The power prediction errors distribution’s probability.

Wind farm	Parameter	Error range (MW)
1^st^	F~N	17.01~26.57
2^nd^	F~N	29.20~39.63
4^th^	F~N	-30.09~-26.06

**Fig 6:**
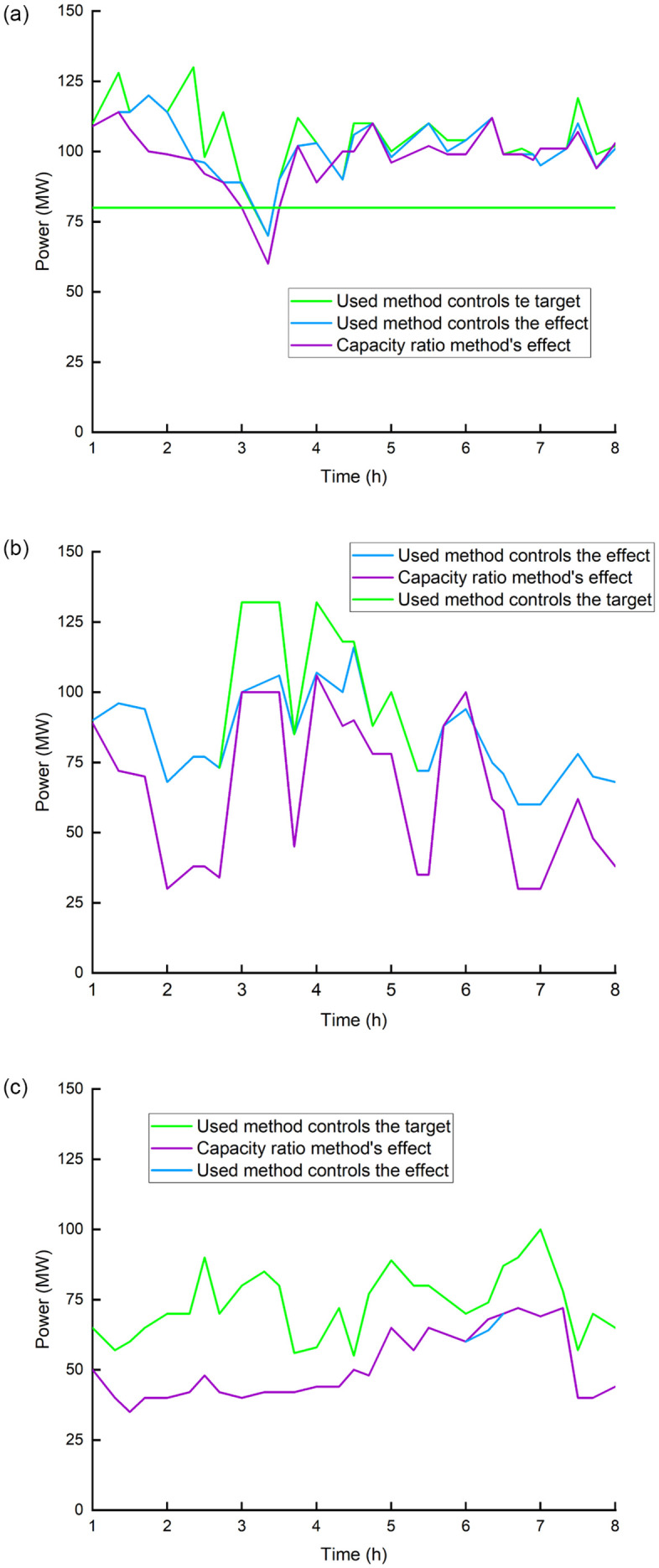
Target and actual value of active power control of each wind farm using proposed method.

In Table 4, the distribution parameters such as F and N value is 22.29 and 24.31 for 1^st^ wind farm, and 34.43 and 26.52 for 2^nd^ wind farm, respectively. The wind power distribution is optimized based on the error distribution and running risk factors. Distribution parameter value of 4^th^ wind farm is -28.23 (F) and 9.33 (N). Furthermore, in comparison to existing control methods that consider errors, in this article we use statistical (more precisely, mean and quantiles) analysis of regional data to classify wind farms based on positive and negative error characteristics, and analyze the confidence in wind farm forecast power. With the predicted power of the wind farm as the input, the confidence interval and expected power of the forecast power are evaluated, and the wind farm’s power control command is optimized. Fig 6 shows the active power control target as well as the theoretically reduced power of certain wind farms.

It can be seen from Fig 6 that due to the negative error characteristic of the 4^th^ wind farm, the probability of failing to meet the control objective is high, and the possible power shortage is transferred to wind farms 1^st^ and 2^nd^. Optimization methods-based control target is near to the actual gain when compared to the capacity ratio method. As started by the proposed method, after 3 hours, the predicted wind power exceeds the transmit guidance. Fig 6a–6c show the control target values for the 1^st^, 2^nd^ and 4^th^ wind farms, respectively. Wind farm 4^th^ produces less electricity than the control goal, which has a higher probability. To maintain a power balance according to the scheduling info, possible power shortages must be transferred to wind farms 1^st^, and 2^nd^, whose probabilities for real power output are higher than the control target. An optimization method is used to distribute the power from three wind farms. The control objectives of the three wind farms are clearly better than those of the capacity proportion technique. Through comparison, it is found that the control deviation can be reduced by 10%, and the capacity proportion method cannot meet the power generation plan.

## 6 Conclusion

Various wind power stations have different distribution characteristics of power forecast errors, which should be taken into account while optimizing and improving active power control of wind farms. The forecast error distribution features of the wind farm are extracted by statistical (more precisely, mean and quantiles) analysis of regional data, and the active power regulation of the wind farm is suitably optimized. The results demonstrate that the proposed method can significantly improve the performance of wind power active power control, and the control deviation in the simulation example can be reduced by about 10%. The model is helpful to reduce the risk of wind power active power control deficiency, which drives from the actual yield being unable to be fulfilled. So as a result of a part of the wind farm’s actual output being lower than the control objective. Our proposed method can effectively reduce the influence of new energy power forecast error on power control, as well as improves the coherence and accuracy of active wind power management.
